# Optical Fiber Fabry–Pérot Microfluidic Sensor Based on Capillary Fiber and Side Illumination Method

**DOI:** 10.3390/s23063198

**Published:** 2023-03-16

**Authors:** Shengnan Wu, Nanfei Lv, Yuhang Geng, Xiaolu Chen, Gaoxuan Wang, Sailing He

**Affiliations:** 1 Ningbo Research Institute, Zhejiang University, Ningbo 315100, China; wushengnan@zju.edu.cn (S.W.);; 2 State Key Laboratory of Modern Optical Instrumentation, College of Optical Science and Engineering, Zhejiang University, Hangzhou 310058, China; 3 School of Information Science and Engineering, NingboTech University, Ningbo 315100, China; 4 South China Academy of Advanced Optoelectronics, South China Normal University, Guangzhou 510006, China; 5 Department of Electrical Engineering, Royal Institute of Technology, SE-100 44 Stockholm, Sweden

**Keywords:** Fabry–Pérot interferometer, capillary fiber, side illumination, microfluidic sensing, temperature compensation

## Abstract

In this paper, an optical fiber Fabry–Pérot (FP) microfluidic sensor based on the capillary fiber (CF) and side illumination method is designed. The hybrid FP cavity (HFP) is naturally formed by the inner air hole and silica wall of CF which is side illuminated by another single mode fiber (SMF). The CF acts as a naturally microfluidic channel, which can be served as a potential microfluidic solution concentration sensor. Moreover, the FP cavity formed by silica wall is insensitive to ambient solution refractive index but sensitive to the temperature. Thus, the HFP sensor can simultaneously measure microfluidic refractive index (RI) and temperature by cross-sensitivity matrix method. Three sensors with different inner air hole diameters were selected to fabricate and characterize the sensing performance. The interference spectra corresponding to each cavity length can be separated from each amplitude peak in the FFT spectra with a proper bandpass filter. Experimental results indicate that the proposed sensor with excellent sensing performance of temperature compensation is low-cost and easy to build, which is suitable for in situ monitoring and high-precision sensing of drug concentration and the optical constants of micro-specimens in the biomedical and biochemical fields.

## 1. Introduction

Microfluidics refers to the liquids and gases that flow in the space at the micron or nanometer level. In the past few decades of fluid sensing research, the microfluidic detection technology has become one of the most important branches in the field [1,2,3,4,5,6]. Among them, refractive index (RI) detection is a property that can be used to infer the properties of fluids. As the most basic property, the RI changes with the concentration of solutes in the solution. Common systems exhibit this behavior, such as saline or aqueous ethanol solution [7,8], etc. Furthermore, the state of biological systems can be inferred from the refractive indices of liquid components, such as glucose dissolved in plasma, which are important for the treatment of diabetes [9,10]. With the expansion of the application scope of microfluidic sensors in the fields of environment, food, medicine and so on [11,12,13], the requirements of social production for microfluidic sensors have gradually increased, such as high sensitivity, high stability, online monitoring, miniaturization, etc.

With the invention of various new optical fibers and the upgrading of optical fiber processing technology, the optical fiber sensors have been deeply studied by an increasing number of scholars, and various optical fiber sensors with new structures or new sensing mechanism have sprung up [7,8,9,10,11,12,13,14,15,16,17,18,19,20,21,22,23,24,25,26]. Due to the superior advantages of small size, high sensitivity, simultaneous measurement of multiple parameters, optical fiber refractive index (RI) sensors have attracted a great deal of attention for physical, chemical and biomedical sensing [1,2,3,4]. Many of these fiber RI sensors are based on the evanescent field interaction with the measured medium, such as long-period gratings (LPG) [14,15,16], fiber Bragg gratings (FBG) [17,18], photonic crystal fibers (PCF) or microstructured fiber [19,20], surface plasmon resonance (SPR) [21,22] and whispering gallery mode (WGM) [23]. However, since most of the light energy is bound in the fiber silica material, sensors based on an evanescent field have disadvantages, such as low RI sensitivity of about tens of nm/RIU [16,17,18] and nonlinear RI response [16]. In previous work, the sensor of open-cavity Fabry–Pérot interferometer (FPI) was found to be able to effectively avoid these limitations [24,25]. The cavity of the FPI sensor can be filled with the RI medium, which causes complete overlap between the light field and the measured medium. The interference fringe of FPI can be used to obtain the optical length of the cavity in linear proportion to the RI of the medium in the cavity. At present, the technologies for fabricating open-cavity FPI microstructures mainly include femtosecond laser etching technology [14,15,16,17,18], special fiber fusion technology [19,20] and selective etching technology [8]. Femtosecond laser beam etching technology forms a microstructure by opening side holes on the fiber [26,27]. However, the requirements of special laser equipment and high-precision machine control often lead to high cost. Special or customized optical fiber fabrication technology is built through optical fiber drawing tower and then manually fused with other optical fibers to form a microstructure [7]. The construction of a fiber drawing tower often leads to high cost and customized fiber fusing processes lead to the bad fabrication consistency. In addition, the selective etching technology uses hydrofluoric acid (HF) solution to chemically etch the optical fiber to form the microstructure [8,28,29]. However, the randomness of hydrofluoric acid (HF) etching often leads to instability of the FPI microstructure, which limits the practical application of FPI based microfluidic sensors. Moreover, almost all of the existing fiber-based microfluidic sensors need an additional microfluidic chip to provide the inlet and outlet of liquid medium [1]. It is a technical challenge for liquid medium to flow into and out of the sensing area conveniently and efficiently [1]. Thus, it is an urgent need to develop a novel, simple, repeatable and low-cost microfluidic fiber sensor technology.

In this paper, a simple hybrid Fabry–Pérot (HFP) microfluidic sensor formed by the capillary fiber (CF) and side illumination method is proposed. Since fabricating without expensive laser machining [14,15,16,17,18], customized fiber fusion or cutting process [8,19,20,28,29], the proposed sensor offers a good performance of reproduction, low-cost and easy fabrication. Taking advantage of the silica cavity formed by the solid silica wall of CF, which is only sensitive to the surrounding temperature, the RI measurement results can be compensated by the common cross-sensitivity matrix method. In addition, instead of an additional microchannel chip, both sides of the CF serve as the inlet and outlet ports. RI and temperature response experiments were both carried out in this study. Specific interference spectrum for different FP cavity can be extracted from the original reflection spectrum by bandpass filtering process. In this study, the silica cavity and the hybrid cavity were selected to analyze and characterize theoretically and experimentally. Experiment results show that the hybrid cavity of the proposed sensor has high microfluidic RI sensitivity. Moreover, the silica cavity is experimentally verified to be only sensitive to the surrounding temperature, which can be used to eliminate the temperature crosstalk. The proposed sensor is suitable for sensing applications of drug concentration and optical constants of micro-specimens in the biomedical and biochemical fields [1,2,3,4].

## 2. Sensing Principle and Fabrication Process

### 2.1. Sensing Principle

**Figure 1:**
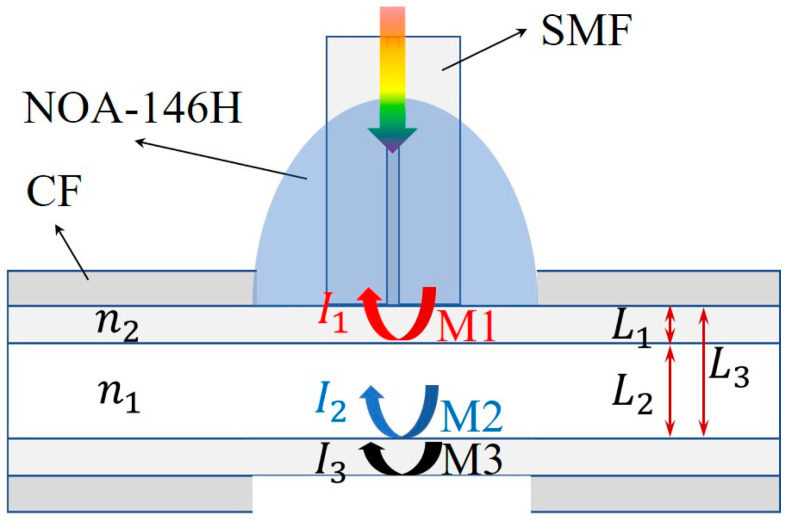
HFP sensor structure schematic.

As shown in Figure 1, the SMF is vertically aligned to one side of CF. To avoid the Fresnel reflection between the contact surface of SMF and CF, an index-matching ultraviolet (UV) glue is used to fill and fix the contact position. Thus, there are three remaining reflection mirrors in our proposed HFP sensor, which are assumed to be M1, M2 and M3, respectively. In addition, as the reflectivities of three reflection mirrors are relatively low according to Fresnel reflection principle, only the first reflection at the cavity mirror is considered in the analysis of reflection spectrum. Thus, the HPF interference can be simplified as a three-beam interference. The reflection spectrum can be expressed as the following [30]:

(1) $$\begin{array}{l} I(\lambda )=I_{1}+I_{2}+I_{3}+2\sqrt{I_{1}I_{2}}\cos(\frac{4\pi n_{1}L_{1}}{\lambda }+\varphi_{12})+2\sqrt{I_{2}I_{3}}\cos(\frac{4\pi n_{2}L_{2}}{\lambda }+\varphi_{23})\\ \;+2\sqrt{I_{1}I_{3}}\cos[\frac{4\pi (n_{1}L_{1}+n_{2}L_{2})}{\lambda }+\varphi_{13}] \end{array},$$

where $I_{1}$, $I_{2}$ and $I_{3}$ are the intensities of the first reflection of the incident light at three different interfaces; $n_{1}$, $n_{2}$ are the RI of the medium inner the CF and the silica wall, respectively; $L_{1}$, $L_{2}$ and $L_{3}$ are the lengths of the silica cavity, hollow cavity and hybrid cavity, respectively; λ is the wavelength of the incident light. The original three-beam reflection interference spectrum of the HPF structure is composed of the reflection spectrums of the hollow cavity, silica cavity and hybrid cavity. The reflection spectrum of specific cavity can be extracted from the original reflection spectrum by a bandpass filtering process. After extraction, the resonant wavelength of reflection spectrum for each cavity can be obtained:

(2) $$\lambda_{r}=\frac{4\pi \sum n_{m}L_{m}}{(2k+1)\pi -\varphi_{0}},$$

where k is an integer which represents the interference order; $L_{m}$, $n_{m}$ are the length and RI of a specific cavity and the integer subscript of m represents different cavity; $\varphi_{0}$ is the initial phase difference between two beams first reflected by two mirrors of a specific FP cavity. It can be seen from Equation (2) that when the RI of the medium and the cavity length changes, the resonant wavelength $\lambda_{r}$ will shift accordingly. The wavelength shift can be expressed as the following:

(3) $$\Delta \lambda_{r}=S_{n}\Delta n+S_{T}\Delta T,$$

where $S_{n}$ and $S_{T}$ are the RI sensitivity and temperature sensitivity, respectively. Hollow cavity is filled with microfluidic medium under testing, which is both sensitive to ambient temperature and medium RI in the CF while the silica cavity is only sensitive to temperature. Since hybrid cavities contain silica cavity and hollow cavity, the hybrid cavity is both sensitive to ambient temperature and medium RI in the CF. Thus, the resonant wavelength shift corresponding to silica cavity, hollow cavity and hybrid cavity toward ambient temperature and medium RI can be respectively expressed as:

(4) $$\begin{array}{l} \Delta \lambda_{hybrid}=S_{n\_hybrid}\Delta n+S_{T\_hybrid}\Delta T\\ \Delta \lambda_{hollow}=S_{n\_hollow}\Delta n+S_{T\_hollow}\Delta T\\ \;\Delta \lambda_{silica}=S_{T\_silica}\Delta T \end{array},$$

where $S_{n\_hybrid}$ and $S_{T\_hybrid}$ are the RI sensitivity and temperature sensitivity of hybrid cavity, respectively; $S_{n\_hollow}$ and $S_{T\_hollow}$ are the RI sensitivity and temperature sensitivity of hollow cavity, respectively; $S_{T\_silica}$ is the temperature sensitivity of silica cavity. All the sensitivities can be further expressed:

(5) $$\begin{array}{l} S_{n\_hybrid}=\frac{4\pi L_{ho}}{(2k+1)\pi -\varphi_{hy}}=\frac{\lambda_{hy}L_{ho}}{n_{ho}L_{ho}+n_{si}L_{si}}\\ S_{T\_hybrid}=\frac{4\pi L_{ho}(\xi_{ho}+n_{ho}\alpha_{ho})}{(2k+1)\pi -\varphi_{hy}}+\frac{4\pi L_{si}(\xi_{si}+n_{si}\alpha_{si})}{(2k+1)\pi -\varphi_{hy}}\\ \;=\frac{\lambda_{hy}[L_{ho}(\xi_{ho}+n_{ho}\alpha_{ho})+L_{si}(\xi_{si}+n_{si}\alpha_{si})]}{n_{ho}L_{ho}+n_{si}L_{si}}\\ S_{n\_hollow}=\frac{4\pi L_{ho}}{(2k+1)\pi -\varphi_{ho}}=\frac{\lambda_{ho}}{n_{ho}}\\ S_{T\_hollow}=\frac{4\pi L_{ho}(\xi_{ho}+n_{ho}\alpha_{ho})}{(2k+1)\pi -\varphi_{ho}}=\frac{\lambda_{ho}(\xi_{ho}+n_{ho}\alpha_{ho})}{n_{ho}}\\ \;S_{T\_silica}=\frac{4\pi L_{si}(\xi_{si}+n_{si}\alpha_{si})}{(2k+1)\pi -\varphi_{si}}=\frac{\lambda_{si}(\xi_{si}+n_{si}\alpha_{si})}{n_{si}} \end{array},$$

where $\lambda_{hy}$, $\lambda_{ho}$ and $\lambda_{si}$ represent the resonant wavelength corresponding to hybrid cavity, hollow cavity and silica cavity of the HFP structure; $L_{hy}$, $L_{ho}$ and $L_{si}$ represent the length corresponding to hybrid cavity, hollow cavity and silica cavity of the HFP structure; α and ξ are the expansion coefficient and the thermo optic coefficient of cavity medium, respectively, which can be written as follows [31,32]:

(6) $$\begin{array}{l} \alpha =\frac{1}{L}\frac{dL}{dT}\\ \xi =\frac{dn}{dT} \end{array}.$$

According to above analysis and equations, once the cavity length and medium are determined, the RI and temperature sensitivities are constants in a specific solution RI range [26,27,28,29]. Therefore, the ambient temperature and medium RI inner the CF can be simultaneously demodulated by any two resonant wavelength shift of silica cavity, hollow cavity or hybrid cavity when the corresponding sensitivities coefficient are measured. The RI difference between microfluidic medium and silica is smaller than the RI difference between air and silica. Thus, silica cavity and hybrid cavity were chosen to study in this work. The variations of ambient temperature and medium RI in the CF can be simultaneously demodulated by solving the cross-sensitivity matrix equation [33]:

(7) ΔTΔn=1ST_silica1ST_hybrid01Sn_hybridΔλsilicaΔλhybrid.

### 2.2. Fabrication Process and FFT Spectra Analysis

**Figure 2:**
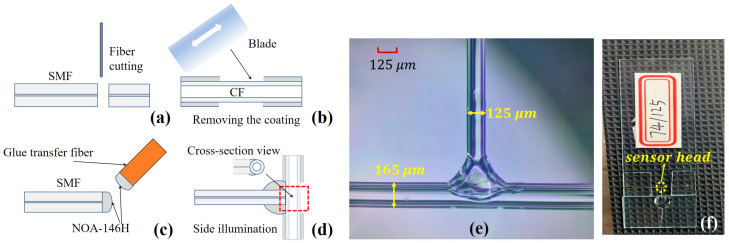
(**a**–**d**) Steps of the sensor fabrication process; (**e**) The microscope image of the fabricated sensor head; (**f**) The sensor fixed on the glass slide.

The fabrication process involved four main steps, as shown in Figure 2a–d. Firstly, a fiber cleaver was used to cut one end of the SMF (Corning, SMF-28e) to make the end surface flat and then fix the SMF on the three-dimensional (3D) adjustment platform; Secondly, a blade was used to manually remove part of the polymer coating layer of the CF (Polymicro technologies, TSP040150, TSP075150 and TSP100200) and then both sides of the CF were clamped on two adjustment platforms. Thirdly, the index-matched UV glue (Norland, NOA-146H) with the RI of ~1.46 RIU (very close to the RI of fused silica) was manually transferred to the flat tail end of SMF through another auxiliary SMF (glue transfer fiber), which can greatly reduce the undesired reflection between the end face of input SMF and the CF. After that, the 3D adjustment platform was used to make the SMF vertically aligned to the CF cladding to form a T-shaped structure (see Figure 2e). During the adjustment, the reflected spectrum of sensor head is observed in real-time to ensure the side aligning quality. After fine-tuning, the centerlines of the SMF and CF are almost located in the same plane by looking for the position with the greatest contrast in the reflection spectrum. Finally, the SMF is vertically side-aligned to the CF and then the UV glue is immediately irradiated with a UV lamp to cure it. It is worth noting that to prevent the upper and lower stress difference of the curing process from causing the two fibers to dislocate, the irradiation direction should be controlled horizontal to the SMF and CF plane.

The microscope image of the fabricated sensor structure is shown in Figure 2e. The hollow cavity of the CF acts as a natural microfluidics channel, which can be conveniently used to detect the RI or concentration of the microfluidics. Fortunately, the naturally solid silica cavity of the CF is only sensitive to ambient temperature which can be used to measure the temperature simultaneously during the microfluidic sensing. In addition, before the calibration experiment, we further verified the mechanical reliability and stability of the UV-curable adhesive fixing point. We pinched the other end of the input SMF and then manually shook the entire sensor sample at a frequency of about 1–2 Hz. The microcavity structure of the sensor sample can still maintain a good connection and there was no obvious change in reflection spectrum. This mainly takes advantage of the good strain resistance of the silica material carrier and the excellent low shrinkage and good optical viscosity of NOA-146H. It should be noted that to further ensure the mechanical stability, the sensor head was fixed on a glass slide by UV glue during the whole experiment (see Figure 2f).

To experimentally study the RI response of the HFP sensor, three HFP sensors formed by different size of CFs were fabricated. The nominal physical parameters of three different types of CF are listed in Table 1, according to CF product manual.

**Figure 3:**
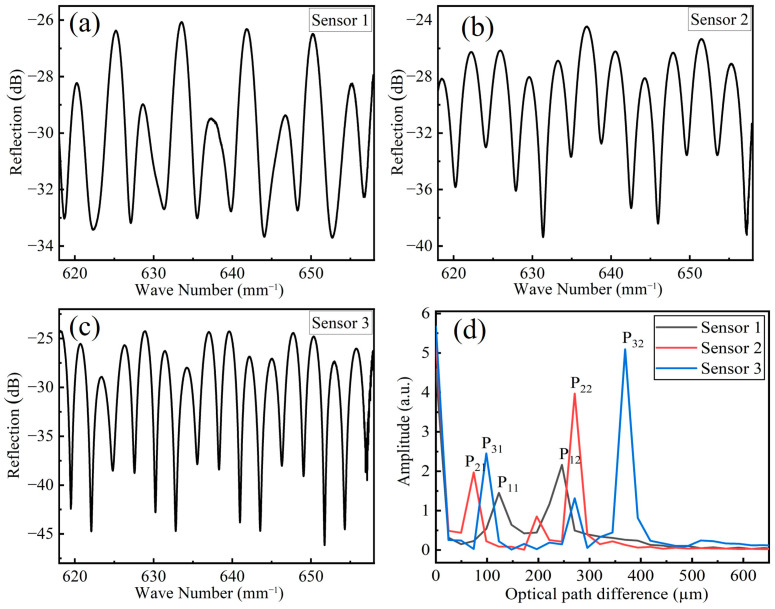
(**a**–**c**) Reflection spectra of three sensors; (**d**) FFT spectra of three sensors.

From Equation (1) we know that the intensity of the reflected light is a trigonometric function of the wavenumber domain 1/λ. Therefore, we firstly performed wavelength reversal on the obtained original reflection spectrum and converted the wavelength domain curve into the wavenumber domain curve. As the reflection spectra after reciprocal processing were not uniformly distributed, we used cubic spline interpolation to increase and uniform the sampling point of the reflection spectra at wavenumber domain. Figure 3a–c are the reflection spectrum of three sensors (S1, S2 and S3) at wave number domain, and Figure 3d is the frequency spectra of three sensors after performing FFT filtering algorithm. In Figure 3d, $P_{11}$, $P_{21}$ and $P_{31}$ are the optical path difference (OPD) corresponding to the silica cavity of three sensors, and their values are 123.12 μm, 73.87 μm and 98.49 µm, respectively; $P_{12}$, $P_{22}$ and $P_{32}$ are the OPD corresponding to the hybrid cavity of three sensors, and their values are 246.23 μm, 270.85 μm and 369.35 μm, respectively. There is a supplementary note that, in the following measuring and data processing, the reflected spectrum of silica cavity or hybrid cavity was extracted by a specific bandpass filter according to the FFT spectra of three sensors. In this study, the 3-dB bandwidth of all the FFT bandpass filters was set at 2 μm.

The RI and temperature sensitivities of these three sensors can be predicted from Equation (5). In this calculation, the medium filled in CF is water, whose RI is n_ho_ = 1.333 and the RI of silica is n_si_ = 1.46. The expansion coefficient and the thermo optic coefficient of silica are α_si_ = 5.5 × 10^−6^/°C and ξ_si_ = 1.178 × 10^−5^/°C, respectively [34,35]. The thermo optic coefficient of deionized water is ξ_water_ = −9.646 × 10^−5^/°C [36]. The resonant wavelength is 1550 nm. Therefore, the theoretical sensitivities of hybrid and silica cavity of three sensors can be calculated and are shown in Table 2.

## 3. Experimental Results and Discussion

### 3.1. Microfludic Refractive Index Response

**Figure 4:**
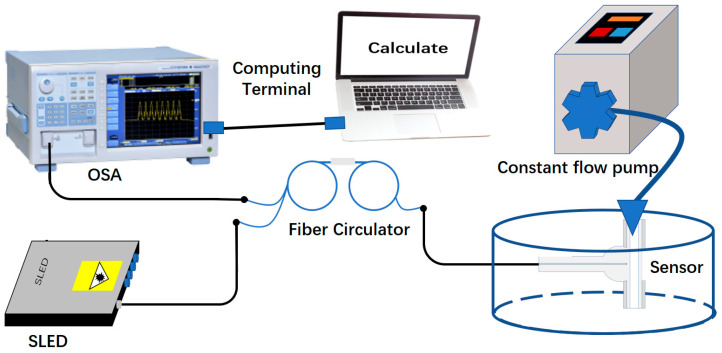
The experimental setup for RI measurement.

The microfluidic RI test setup is schematically shown in Figure 4. The devices include: a broadband light source (DL-BX9-CS5169A, Denselight, Singapore), an optical spectrum analyzer (OSA, AQ6370D, YOKOGAWA, Japan), a fiber optic circulator and a microfluidic pump (LD-P2020, Shanghai Lande Co., Ltd., Shanghai, China). The broadband light source provides an output light with wavelength range of 1500–1600 nm. In this microfluidic RI experiment, the potassium chloride (KCl) solutions with RI in the range of 1.3374 to 1.3490 were prepared in advance and were injected in steps into the CF as microfluidic samples to characterize the refractive index response of the sensors. The concentration of KCl solution and the corresponding RI value are measured with the Abbe refractometer (WAY-2WAJ, Zhejiang LICHEN Instrument Technology Co., Ltd., Shaoxing, China) and shown in Table 3. Here, the refractive indexes corresponding to the employed solutions all refer to their values at the wavelength of 589.3 nm and at room temperature.

Firstly, the KCl solution was slowly injected into the hollow channel of the CF of HFP sensor through a microfluidic pump. One end of the CF was glued to the output plastic pipe of the microfluidic pump with UV (NOA-61, Norland) sealing. After ensuring that the solution fills the cavity, the injection was stopped and left for a period to prevent the influence of the microfluidic flow on the experimental results, after which the original reflection spectrum of the HFP sensor at different RIs were recorded by using the OSA. Before injecting KCl solution with another concentration, the microfluidic channel of CF was rinsed with deionized water for about 5 min. The concentration of the KCl solution was increased from 2% to 10% with increments of 2% and the above operation was repeated until the reflection spectra of three HFP sensors in the experiment were recorded.

It should be noted that as a result of the small RI difference between silica and solution, the interference amplitude corresponding to the hollow cavity of the HFP sensor is weaker when CF is filled with solution. Therefore, we chose the hybrid cavity and silica cavity to detect the microfluidic RI and temperature. Two different band-pass filters with bandwidth of 0.01 μm were set to extract the interference spectrum of the hybrid cavity and the silica cavity in the original reflection spectrum.

**Figure 5:**
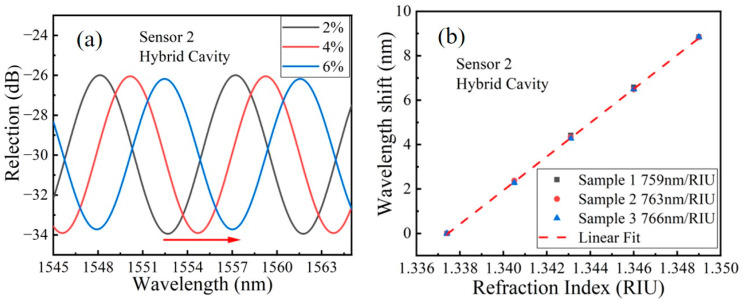
(**a**) The extracted interference spectrums of the hybrid cavity of S2; (**b**) The resonant wavelength drift with different RI of three samples of S2 fabricated at different batch.

Taking advantage of the natural uniform size of CF, one advantage of our proposed HFP sensor head is the good performance of reproduction. Figure 5a shows the extracted interference spectrums of the hybrid cavity of S2. When the RI continues to increase, the resonant wavelength of the interference spectrum drifts toward the long wavelength direction. Figure 5b shows the resonant wavelength drift with different RIs of three samples of S2 fabricated at a different batch. The linear fitting result of Figure 5b shows that the RI sensitivities of three samples of S2 are 759 nm/RIU, 763 nm/RIU and 766 nm/RIU, respectively. In this study, the RI response repeatability of different batches of the HFP sensor is defined as the ratio of maximum sensitivities errors. The sensitivities mean and the ratio is about 0.9% for S2, which indicated that the proposed HFP sensor possesses excellent refractive index response repeatability.

**Figure 6:**
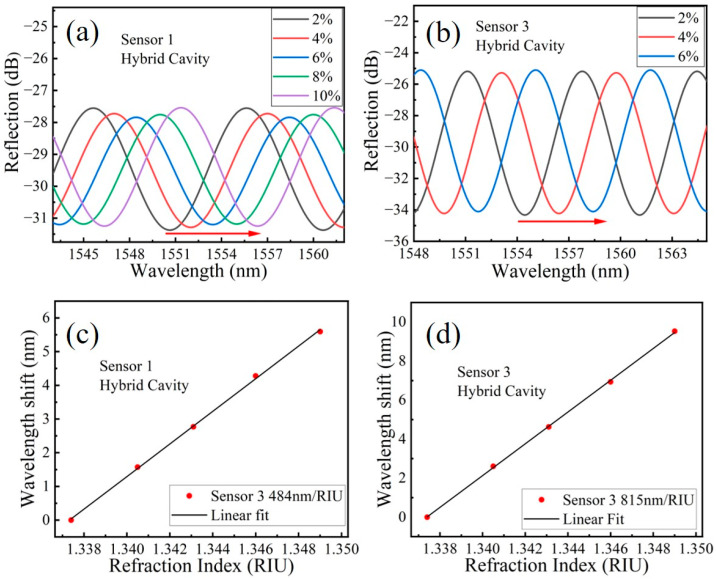
(**a**,**b**) The extracted interference spectrums of the hybrid cavity of S1 and S3, respectively; (**c**,**d**) The resonant wavelength drift with different RI of S1 and S3, respectively.

Subsequently we also tested the RI responses of sensors 1 and 3, the results of which are shown in Figure 6. From Figure 6c,d, the RI sensitivities of sensors 1 and 3 are 484 nm/RIU and 815 nm/RIU, respectively. From the experimental results of RI test, it can be found that the measured RI sensitivities of three sensors basically coincide with the theoretical value shown in Table 2 and the difference may be caused by the deviation of practical size of CF and RI of silica. In addition, from the interference spectrum shown in Figure 5a and Figure 6a,b, the contrast ratios of the hybrid cavity interference spectrum of S1, S2 and S3 are about 3.8 dB, 7.9 dB and 9.1 dB, respectively, and the free spectral range (FSR) of the hybrid cavity of S1, S2 and S3 are about 9.6 nm, 9.1 nm and 7.56 nm, respectively. Considering both with the RI sensitivity and the FSR, the appropriate cavity length can be flexibly selected when the HFP sensor is used for practical microfluidic detection.

### 3.2. Temperature Response

**Figure 7:**
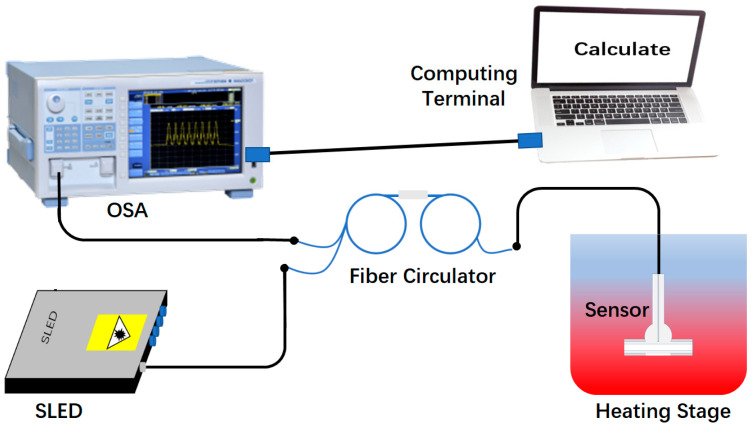
The experimental setup for temperature measurement.

**Figure 8:**
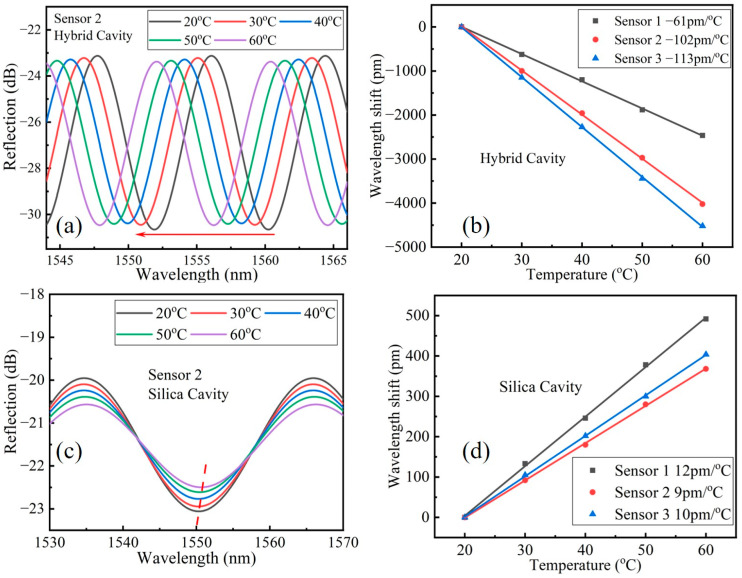
(**a**,**c**) The extracted spectra of the hybrid cavity and silica cavity of S2 under different temperature, respectively; (**b**,**d**) The linear fitting of resonant wavelength shift of the hybrid cavity and silica cavity toward temperature of S1, S2 and S3, respectively.

We next performed temperature sensing on the HFP sensors, and the setup of microfluidic temperature test is schematically shown in Figure 7. The model of OSA, broadband light source and fiber circulator used here were the same as the case in RI test experiment. A water bath heating stage (HH-11-1, Shanghai Zhulan Co., Ltd., Shanghai, China) was used to produce different ambient temperatures. All the experimental setup was placed on the optical platform to avoid the influence of environmental vibration. Moreover, it should be noticed that the CF was filled with deionized water during the temperature test. In the experiment, the sensor was placed on the heating platform, and then the temperature of the heating platform was adjusted from 20 °C to 60 °C with a step of 10 °C. At each temperature setting points, we kept the temperature constant for 20 min before recording the spectrum to ensure the accuracy of the measurement results. Repeating such steps, the reflectance spectrum of the three sensors at five different temperatures were recorded. The results are shown in Figure 8.

Figure 8a,c are the spectrum extracted by the hybrid cavity and silica cavity of S2 at different temperatures. We found that as the temperature gradually increases, the resonant wavelength of the silica cavity red shifted while the resonant wavelength of the hybrid cavity blue shifted. This can be attributed to the positive thermo-optical coefficient and thermal expansion coefficient of silica material and the large negative thermo-optical coefficient of KCl solution. Figure 8b,d are the linear fitting of resonant wavelength shifts of the hybrid cavity and silica cavity toward temperature of S1, S2 and S3, respectively. The temperature sensitivities of hybrid cavity of S1, S2 and S3 are −61 pm/°C, −102 pm/°C and −113 pm/°C, respectively, and the temperature sensitivities of silica cavity of S1, S2 and S3 are 12 pm/°C, 9 pm/°C and 10 pm/°C, respectively. It can be seen that though the measured temperature sensitivities of three sensors had a slight deviation to the theoretical value shown in Table 2, the sensitivities variation trend of three sensors was consistent with the theoretical value, which indicated that the difference was mainly caused by the deviation of practical thermal optics coefficient of KCl solution and the deviation of practical thermal optics and thermal expansion coefficient of silica.

Therefore, according to the calibration results shown in Figure 5, Figure 6 and Figure 8, the microfluidic RI variation Δn and temperature variation ΔT can be simultaneously determined by solving the cross-sensitivity matrix Equation (7). Taking S2 as an example, the microfluidic RI sensitivity S_n_hybrid_ is 763 nm/RIU and the temperature sensitivity S_T_hybrid_ and S_T_silica_ of the hybrid cavity and silica cavity are −102 pm/°C and 9 pm/°C, respectively. By putting the sensitivity parameters into Equation (7), we can get:

(8) $$\left[\begin{array}{c} \Delta T\\ \Delta n \end{array}\right]=\left[\begin{array}{cc} 0.1111{}^{\circ}C/\mathrm{pm} & -0.0098{}^{\circ}C/\mathrm{pm}\\ 0 & 0.0013/\mathrm{nm} \end{array}\right]\left[\begin{array}{c} \Delta \lambda_{silica}\\ \Delta \lambda_{hybrid} \end{array}\right],$$

A comparison of various representative fiber RI sensors with different optical configurations previously reported is listed in Table 4. It should be noted that the item ‘Sensing Length’ only represents the length of the sensitive area and does not include the overall fiber and package length. As seen in Table 4, fiber RI sensors based on the evanescent field interaction with the measured medium yield a low sensitivity and temperature crosstalk generally cannot be eliminated without hybrid structure [16,18,23]. In our previous work, highly sensitive two-parameter measurements have been achieved by a dual SPR sensor [22]. However, magnetron sputtering and chemical etching is required and the sensing length is still not compact enough. Hybrid FPI sensing structure can achieve compact size and temperature compensation [25], whereas most of the existing hybrid FPI structure need customized fiber cutting and fusion operation. In this work, a hybrid FPI based on a CF and a simple side illumination method can realize a high RI sensitivity, temperature elimination and compact size at the same time. Moreover, the CF can act as a natural microchannel without any complex processing, such as chemical etching, lasering writing or machining and customized fiber operation etc.

## 4. Conclusions

In summary, a hybrid FPI sensor structure based on a CF and a simple side illumination method has been proposed, fabricated and characterized. Theoretical and experimental studies verified that the proposed sensor can simultaneously measure microfluidic RI and temperature by solving the cross-sensitivity matrix equation while possessing a good repeatability performance. The experimental results show that the HFP sensors have an excellent linear RI response with a high sensitivity of 484 nm/RIU, 763 nm/RIU and 815 nm/RIU for hybrid cavity of S1, S2 and S3, respectively, and an excellent linear temperature response with a sensitivity of 12 pm/°C, 9 pm/°C and 10 pm/°C for silica cavity of S1, S2 and S3, respectively. The RI response repeatability of different batch of the HFP sensor in terms of the ratio of maximum sensitivities errors and the sensitivities mean and the ratio is about 0.9% for S2. Compared with most existing fiber-optic microfluidic sensors, the proposed HFP sensor has the advantages of high RI sensitivity and ability to simultaneously measure RI and temperature and good reproduction performance, which make it suitable for practical microfluidic RI and temperature simultaneous monitoring.

## Figures and Tables

**Table 1:** Physical parameter table of three types of CF.

Arguments	CF 1	CF 2	CF 3
Fiber model	TSP040150	TSP075150	TSP100200
Hollow diameter	40 µm	74 µm	101 µm
Cladding diameter	126 µm	125 µm	167 µm

**Table 2:** Theoretical sensitivities of three sensors.

Theoretical Sensitivity	S1	S2	S3
S_n_hybrid (nm/RIU)	534.0	844.2	851.6
S_T_hybrid (pm/°C)	−36.2	−69.5	−70.3
S_T_silica (pm/°C)	21.0	21.0	21.0

**Table 3:** The relationship between the concentration and the RI of KCl solutions.

The Concentration of the Solution	The RI of the Solution
2%	1.3374
4%	1.3405
6%	1.3431
8%	1.3460
10%	1.3490

**Table 4:** Performance comparison with previously reported fiber RI sensors.

References	Schematic	Fabrication Technique	Sensing Length	RI Sensitivity	Temperature Compensation
[18]	Etched FBG	Chemical etching	2.5 mm	92 nm/RIU	no
[16]	Dual LPG based MZI	Excimer laser writing	40 mm	58.8 nm/RIU	no
[23]	WGM	Fiber tapering	~100 µm	174.7 nm/RIU	no
[22]	Dual SPR	Magnetron sputtering	10 mm	2015.5 nm/RIU	yes
[25]	Hybrid FPI based on C shape fiber	Customized fiber operation	~125 µm	1704 nm/RIU	yes
This work	Hybrid FPI based on capillary fiber	Side illuminating	~125 µm	815 nm/RIU	yes

## Data Availability

Not applicable.

## References

[1] Li L., Zhang Y., Zhou Y., Zheng W., Sun Y., Ma G., Zhao Y. (2021). Optical Fiber Optofluidic Bio-Chemical Sensors: A Review. Laser Photonics Rev..

[2] Pissadakis S. (2019). Lab-in-a-fiber sensors: A review. Microelectron. Eng..

[3] Jahanbakhsh A., Wlodarczyk K., Hand D., Maier R., Maroto-Valer M. (2020). Review of Microfluidic Devices and Imaging Techniques for Fluid Flow Study in Porous Geomaterials. Sensors.

[4] Zhao Y., Hu X., Hu S., Peng Y. (2020). Applications of fiber-optic biochemical sensor in microfluidic chips: A review. Biosens. Bioelectron..

[5] Wu R., Kim T. (2021). Review of microfluidic approaches for fabricating intelligent fiber devices: Importance of shape characteristics. Lab Chip.

[6] Zhao X., Zhou Y., Li Y., Guo J., Liu Z., Luo M., Guo Z., Yang X., Zhang M., Wang Y. (2022). Ultrasensitive optofluidic coupled Fabry–Perot capillary sensors. Opt. Express.

[7] Wu C., Liu Z., Zhang A., Guan B., Tam H. (2014). In-line open-cavity Fabry–Pérot interferometer formed by C-shaped fiber fortemperature-insensitive refractive index sensing. Opt. Express.

[8] Wu S., Yan G., Zhou B., Lee E.-H., He S. (2015). Open-Cavity Fabry-Perot Interferometer Based on Etched Side-Hole Fiber for Microfluidic Sensing. IEEE Photonics Technol. Lett..

[9] Liao W., Tu Y., Wu M., Lin J., Wang H., Chien K. (2015). Blood glucose concentration and risk of pancreatic cancer: Systematic review and dose-response meta-analysis. Br. Med. J..

[10] Tan Q., Wu S., Liu Z., Wu X., Forsberg E., He S. (2022). High sensitivity detection of SARS-CoV-2 by an optofluidic hollow eccentric core fiber. Biomed. Opt. Express.

[11] Yager P., Edwards T., Fu E., Helton K., Nelson K., Tam M.R., Weig B.H. (2006). Microfluidic diagnostic technologies for global public health. Nature.

[12] Song H., Chen D., Ismagilov R. (2010). Reactions in Droplets in Microfluidic Channels. Angew. Chem. Int. Ed..

[13] Beebe D., Mensing G., Walker G. (2002). Physics and applications of microfluidics in biology. Annu. Rev. Biomed. Eng..

[14] Han M., Guo F., Lu Y. (2010). Optical fiber refractometer based on cladding-mode Bragg grating. Opt. Lett..

[15] Rindorf L., Jensen J.B., Dufva M., Pedersen L.H., Hoiby P.E., Bang O. (2006). Photonic crystal fiber long-period gratings for biochemical sensing. Opt. Express.

[16] Fan Y., Zhu T., Shi L., Rao Y. (2011). Highly sensitive refractive index sensor based on two cascaded special long-period fiber gratings with rotary refractive index modulation. Appl. Opt..

[17] Bandyopadhyay S., Dey T.K., Basumallick N., Biswas P., Dasgupta K., Bandyopadhyay S. (2016). High Sensitive Refractometric Sensor Using Symmetric Cladding Modes of an FBG Operating at Mode Transition. J. Light. Technol..

[18] Lee S., Jeong M., Saini S. (2012). Etched-Core Fiber Bragg Grating Sensors Integrated with Microfluidic Channels. J. Light. Technol..

[19] Pu M., Liu L., Frandsen L.H., Ou H., Yvind K., Hvam J.M. (2010). Silicon-on-Insulator Ring-Shaped Photonic Crystal Waveguides for Refractive Index Sensing. Natl. Fiber Opt. Eng. Conf..

[20] Rindorf L., Hoiby P.E., Jensen J.B., Pedersen L.H., Bang O., Geschke O. (2006). Towards biochips using microstructured optical fiber sensors. Anal. Bioanal. Chem..

[21] Gauvreau B., Hassani A., Fehri M., Kabashin A., Skorobogatiy M. (2007). Photonic bandgap fiber-based Surface Plasmon Resonance sensors. Opt. Express.

[22] Wu S., Tan Q., Forsberg E., Hu S., He S. (2020). In-situ dual-channel surface plasmon resonance fiber sensor for temperature-compensated detection of glucose concentration. Opt. Express.

[23] Niu P., Jiang J., Liu K., Wang S., Wang T., Liu Y., Zhang X., Ding Z., Liu T. (2022). High-sensitive and disposable myocardial infarction biomarker immunosensor with optofluidic microtubule lasing. Nanophotonics.

[24] Wang Y., Wang D.N., Liao C.R., Hu T., Guo J., Wei H. (2013). Temperature-insensitive refractive index sensing by use of micro Fabry–Pérot cavity based on simplified hollow-core photonic crystal fiber. Opt. Lett..

[25] Li X., Warren-Smith S.C., Xie L., Ebendorff-Heidepriem H., Nguyen L.V. (2020). Temperature-Compensated Refractive Index Measurement Using a Dual Fabry–Perot Interferometer Based on C-Fiber Cavity. IEEE Sens. J..

[26] Tian M., Lu P., Chen L., Liu D., Yang M. (2013). Micro Multicavity Fabry–Pérot Interferometers Sensor in SMFs Machined by Femtosecond Laser. IEEE Photonics Technol. Lett..

[27] Zhang C., Fu S., Tang M., Liu D. Femtosecond Laser Fabricated All-Multicore-Fiber Parallel Fabry-Perot Interferometers for Dual-Parameter Sensing. Proceedings of the Optical Fiber Communications Conference and Exhibition (OFC).

[28] Preter E., Preloznik B., Artel V., Sukenik C.N., Donlagic D., Zadok A. (2013). Monitoring the Evaporation of Fluids from Fiber-Optic Micro-Cell Cavities. Sensors.

[29] Pevec S., Donlagic D. (2014). High resolution, all-fiber, micro-machined sensor for simultaneous measurement of refractive index and temperature. Opt. Express.

[30] Wu Y., Zhang Y., Wu J., Yuan P. (2017). Fiber-optic hybrid-structured Fabry–Perot interferometer based on large lateral offset splicing for simultaneous measurement of strain and temperature. J. Light. Technol..

[31] Zhao Y., Zhang Y. (2014). Research on temperature and magnetic field sensing characteristics of photonic crystal fiber filled with magnetic fluid. Microw. Opt. Technol. Lett..

[32] Tian J., Jiao Y., Ji S., Dong X., Yao Y. (2018). Cascaded-cavity Fabry–Perot interferometer for simultaneous measurement of temperature and strain with cross-sensitivity compensation. Opt. Commun..

[33] Liu Y., Zhang T., Wang Y., Yang D., Liu X., Fu H., Jia Z. (2018). Simultaneous measurement of gas pressure and temperature with integrated optical fiber FPI sensor based on in-fiber micro-cavity and fiber-tip. Opt. Fiber Technol..

[34] Yang N., Qiu Q., Su J., Shi S. (2014). Research on the temperature characteristics of optical fiber refractive index. Optik.

[35] Lee C., Ho H., Gu J., Yeh T., Tseng C. (2015). Dual hollow core fiber-based Fabry–Perot interferometer for measuring the thermo-optic coefficients of liquids. Opt. Lett..

[36] Kim Y.H., Park S.J., Jeon S.W., Ju S., Park C.S., Han W.T., Lee B.H. (2012). Thermo-optic coefficient measurement of liquids based on simultaneous temperature and refractive index sensing capability of a two-mode fiber interferometric probe. Opt. Express.

